# Real-Time Automated Measurements of Optic Nerve Sheath Diameter for Noninvasive Assessment of Intracranial Pressure in Aneurysmal Subarachnoid Hemorrhage

**DOI:** 10.1007/s12028-024-02194-w

**Published:** 2025-01-07

**Authors:** Dag Ferner Netteland, Mads Aarhus, Else Charlotte Sandset, Angelika Sorteberg, Llewellyn Padayachy, Eirik Helseth, Reidar Brekken

**Affiliations:** 1 Department of Neurosurgery, Oslo University Hospital, Oslo, Norway; 2 Faculty of Medicine, University of Oslo, Oslo, Norway; 3 Department of Neurology, Oslo University Hospital, Oslo, Norway; 4 The Norwegian Air Ambulance Foundation, Oslo, Norway; 5 Department of Neurosurgery, Faculty of Health Sciences, University of Pretoria, Steve Biko Academic Hospital, Pretoria, South Africa; 6 Department of Health Research, Medical Technology, SINTEF, Trondheim, Norway

**Keywords:** Noninvasive, Ultrasound, Optic nerve sheath, Intracranial pressure, Aneurysmal subarachnoid hemorrhage

## Abstract

**Background:**

Optic nerve sheath diameter (ONSD) is a promising noninvasive parameter for intracranial pressure (ICP) assessment. However, in the setting of aneurysmal subarachnoid hemorrhage (aSAH), several previous studies have reported no association between ultrasonically measured ONSD and ICP. In this study, we evaluate ONSD in patients with aSAH using a novel method of automated real-time ultrasonographic measurements and explore whether factors such as having undergone surgery affects its association to ICP.

**Methods:**

We prospectively included adult patients with aSAH undergoing invasive ICP monitoring. ONSD was obtained using a prototype ultrasound machine with software for real-time automated measurements at the bedside. Correlation between ONSD and ICP was explored, and the ability of ONSD to discriminate dichotomized ICP was evaluated. Abovementioned analyses were performed for the whole cohort and repeated for subgroups by whether the basal cisterns had been surgically entered before ultrasound examination.

**Results:**

Twenty-six ultrasound examinations were performed in 20 patients. There was a positive correlation between ONSD and ICP (*R* = 0.43; *p* = 0.03). In the subgroup where the basal cisterns had not been surgically entered before ultrasound examination, there was a stronger correlation (*R* = 0.55; *p* = 0.01), whereas no correlation was seen in the subgroup where the basal cisterns had been surgically entered (*R* = − 0.16; *p* = 0.70). ONSD displayed an ability to discriminate ICP dichotomized at ≥ 15 mm Hg (area under the curve [AUC] = 0.84, 95% confidence interval [CI] 0.65–0.96). Subgroup analysis revealed a perfect discriminatory ability (AUC = 1, 95% CI 0.81–1) where the basal cisterns had not been surgically entered and no discriminatory ability (AUC = 0.47, 95% CI 0.16–0.84) where the basal cisterns had been surgically entered before ultrasound examination.

**Conclusions:**

Automatically measured ONSD correlated well with ICP and displayed a perfect discriminatory ability in patients with aSAH in whom the basal cisterns had not been entered surgically before ultrasound examination, and may be a clinically valuable noninvasive marker of ICP in these patients. Caution should be exercised in using ONSD in patients in whom the basal cisterns have been entered surgically before ONSD measurements, as no association was observed in this subgroup.

**Supplementary Information:**

The online version contains supplementary material available at 10.1007/s12028-024-02194-w.

## Introduction

Aneurysmal subarachnoid hemorrhage (aSAH) is characterized by an abrupt arterial bleed into the subarachnoid space and a concurrent abrupt rise in intracranial pressure (ICP) [1, 2]. Secondary rises in ICP may result from hydrocephalus and brain swelling related to cerebral hypoperfusion [3]. Intracranial hypertension may itself cause, or exacerbate, cerebral hypoperfusion by decreasing cerebral perfusion pressure. ICP monitoring to enable detection and management of intracranial hypertension is therefore an integral part of the management of the condition in many centers [4].

Today, the standard mode of ICP assessment remains invasive. However, invasive ICP measurement is demanding on resources and recent data from SYNAPSE-ICU [4] suggests large discrepancies in the routine use of ICP monitors globally, with considerably lower rates in surveyed centers in low/middle income countries (LMIC) as compared with high-income countries (HIC). Discrepancies were present throughout underlying pathologies and included aSAH for which the reported rates of routine use were more than double in HIC versus LMIC (67% in HIC, 29% in LMIC).

Against this background, a quick, reliable, and cost-effective noninvasive alternative would clearly be desirable. Optic nerve sheath diameter (ONSD) is a promising noninvasive parameter for ICP assessment. Embryologically starting out as outpouchings of the central nervous system, the optic nerves are surrounded by cerebral spinal fluid (CSF) enveloped by meningeal sheaths. ICP can therefore propagate in the CSF along the optic nerves and distend the optic nerve sheath, and its diameter can be measured, for example, using bedside ultrasonography.

An association between ONSD and ICP has been firmly established in traumatic brain injury and in mixed patient populations [5]. In the setting of aSAH, however, an association between ONSD and ICP has not yet been clearly established. On the contrary, results are diverging, and although an association has been reported in aSAH subgroups of studies with mixed populations [6, 7], several studies of pure aSAH cohorts have reported no correlation between ultrasonically measured ONSD and invasively measured ICP [8, 9]. Proposed reasons for this have included that the sudden and substantial rise in ICP associated with the initial bleed may lead to irreversible distention of the optic nerve sheath, precluding its subsequent use as a parameter sensitive to further ICP changes [8–10]. Another is that the perineural subarachnoid space is trabeculated and may be clotted by blood products, thereby losing its ability to propagate the increased CSF pressure along the nerve [8, 11].

In this study, we examined the association between ONSD and ICP in a cohort of aSAH patients using automated real-time ultrasonographic measurements of the optic nerve sheath at the bedside and explored whether factors such as having undergone surgery may affect this association.

## Methods

In this study evaluating the ability of automatically measured ONSD to assess ICP, we prospectively included adult patients with aSAH who were admitted to Oslo University Hospital between September 2022 and June 2024. Invasive ICP measurements served as the reference to which ONSD was compared. Patients were examined by transorbital ultrasound and thereby included in the study, as per availability of the ultrasound operator.

In accordance with the Helsinki Declaration [12], proxy consent was obtained for unconscious patients at the time of inclusion. If the patient regained ability to give informed consent, this was obtained from the patient at a later stage. The study was approved by the Regional Committee for Medical and Health Research Ethics South East Norway (2019/811).

### Patient Eligibility

Adult patients (≥ 18 years old) admitted to Oslo University Hospital with an imaging-based diagnosis of aSAH and who were invasively ICP monitored were eligible for inclusion. Invasive ICP monitoring was required to be recorded via a correctly positioned and functioning parenchymal sensor or an external ventricular drain.

### Data Acquisition

Transorbital ultrasound examination was performed by a single experienced operator (DFN) using a CE-marked prototype ultrasound scanner with a linear array probe and software (Nisonic P-100 ultrasound scanner, Nisonic AS, Trondheim, Norway) for real-time automated ONSD measurements at the bedside. Established safety margins of ophthalmic ultrasound imaging were adhered to, with a mechanical index of less than 0.23. Serial measurements in the same patient were obtained at opportunity.

Invasive ICP was measured using either a parenchymal microsensor (Raumedic Neurovent-P ICP sensor; Raumedic AG, Münchberg, Germany) or a ventricular catheter and was recorded at the time of the ultrasound examination.

Additional parameters, including details of patient demographics, clinical presentation, SAH and aneurysm characteristics, physiological parameters, and management including the presence of CSF drainage and the method of aneurysm repair, as well as the nonsurgical management, were recorded. Times of ictus, admission, and treatment were also gathered. When ictus was unknown, the time when the patient was last known to be well was used.

### Software for Automated ONSD Measurements

**Fig. 1:**
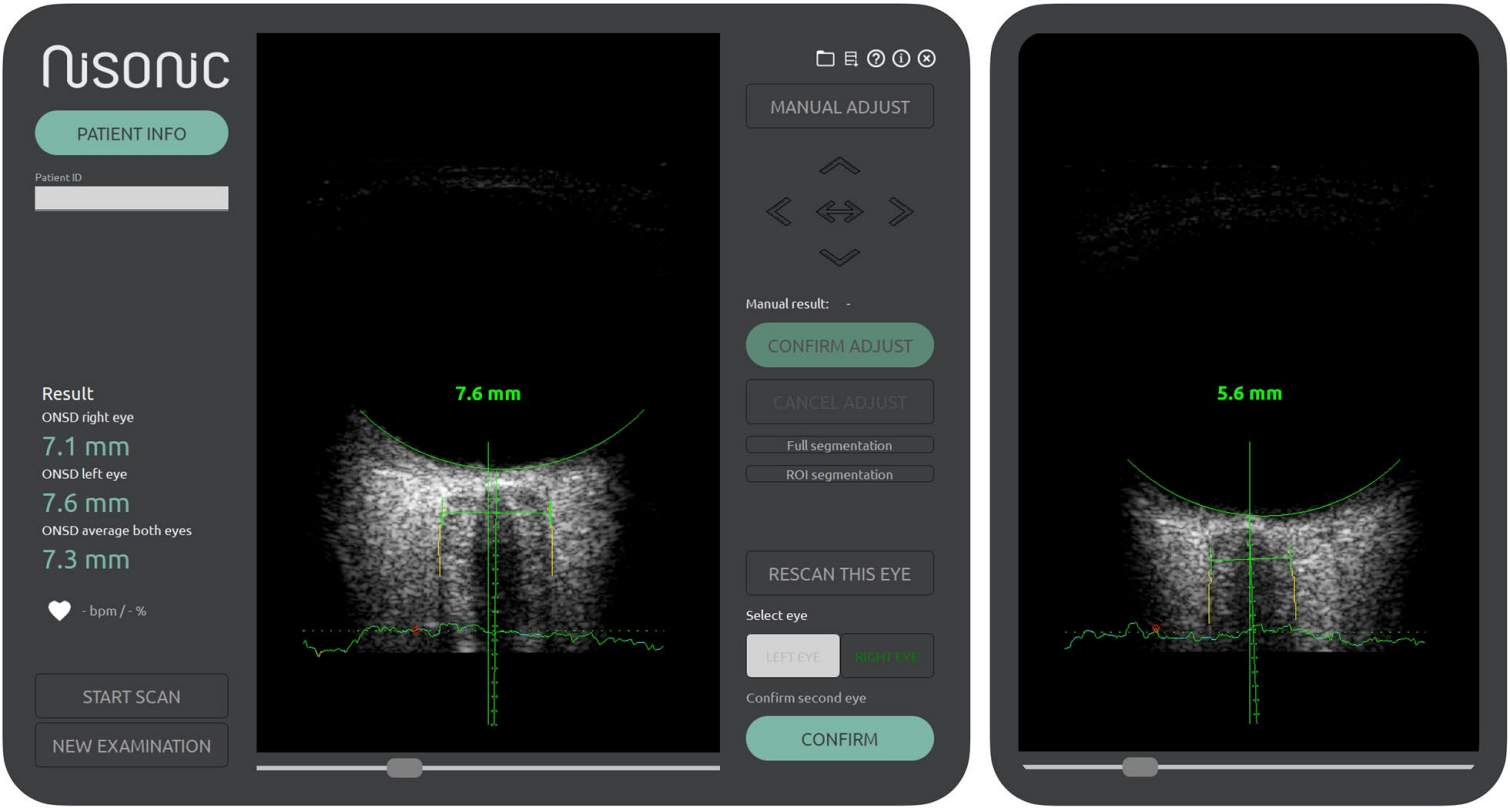
Left: example of an ultrasound image with the software interface during optic nerve sheath examination; right: second example ultrasound image with the surrounding software interface removed. In the ultrasound images the yellow lines along the optic nerve sheath represents the real-time segmentation of this structure. The horizontal green line connecting them represents the real-time optic nerve sheath diameter measurement 3 mm behind the retina. For the software guidance system, color coded graphics are used where green, yellow, and red annotates good, intermediate and poor, respectively. Green and yellow are accepted by the software while red is unacceptable thereby halting processing. For image alignment the software uses two graphics, first the curved line along the retina, which in these examples are green (good). Second, the stippled lines along the nerve axis, which in the examples are also coded green, due to the good image alignment with the nerve axis. The horizontal lines along the bottom indicate the probe movement during the 10-s recording sequence

The software is implemented as an integral part of the prototype ultrasound machine (NiSonic P-100) and provides a guidance system, as previously described by our group [13], to aid the operator in appropriate image alignment with the optic nerve axis. It also indicates the amount of probe movement. The software automatically records an image sequence of the optic nerve sheath in one plane once image alignment and probe movements are within defined thresholds over a 10-s period. In real time, the optic nerve sheath is automatically segmented, and the ONSD is automatically calculated by the software using the 75th percentile of the measured diameters over the entire image sequence (10 s). An image of the user interface during image acquisition as well as two examples of the nerve sheath segmentation for two different diameters are provided in Fig. 1. The software allows for manual corrections of the optic nerve sheath segmentation at the clinician’s wish. In this study, all ONSD measurements were solely based on the automatic segmentation, without the use of manual correction, to standardize the measurements and to test the performance of the software.

### SAH Management

Included patients were treated according to our institutional aSAH management protocol, and inclusion in the study did not interfere with standard management [14]. As per this protocol, maintaining ICP ≤ 20 mm Hg is a main goal of management. CSF drainage via an external ventricular drain (EVD) in the acute phase represents a cornerstone of the protocol to manage post-SAH hydrocephalus and to control ICP and maintain an adequate cerebral perfusion pressure (CPP). Nonsurgical ICP lowering measures include elevation of head of bed to 30°, deep sedation, hypertonic saline, normocapnia, and thermoregulation to normothermia. Last-tier measures include decompressive craniectomy and thiopental sedation. Early aneurysm repair is aimed for and is by either endovascular or surgical techniques [15], based on a case-to-case multidisciplinary discussion.

### End Points and Subgrouping by Surgical Entrance to the Basal Cisterns

Prespecified end points were (1) correlation between ONSD and ICP and (2) the ability of ONSD to discriminate dichotomized ICP.

To further explore potential physiological reasons for correlations between ONSD and ICP, or the lack thereof, the same analyses were repeated after subgrouping based on whether the basal cisterns were surgically entered while securing the aneurysm by clip ligation. This was based on observations that ONSD tended to be large in patients who had undergone surgery with entrance of the basal cisterns, despite ICP being low. These observations, in turn, led to a hypothesis that the opening of arachnoid CSF divisions in the basal cisterns may facilitate the CSF flow, and hence the fluid pressures, that are propagated into the perioptic cisterns, thereby disturbing the baseline premises of ONSD.

In the subgroup of patients in whom the basal cisterns were surgically entered before ultrasound examination, we included patients who had undergone clip ligation of an aneurysm in the anterior circle of Willis (including posterior communicating artery) or if the anterior circle of Willis was exposed as part of gaining proximal control in more distal aneurysms. The subgroup of patients in whom the basal cisterns were not surgically entered before ultrasound examination included patients who had undergone endovascular aneurysm repair and patients who had undergone clip ligation of more distal aneurysms (e.g., middle cerebral artery aneurysms) without entrance to the basal cisterns during the surgical procedure.

### Data Analysis

Correlation between ONSD and ICP was explored using Pearson correlations, and results were reported by correlation coefficients *R*. Linear regression models with ONSD as the independent variable and ICP as the dependent were fitted where correlations indicated the strongest associations. Assumptions for Pearson correlations and linear regressions were evaluated, including the presence of outliers for both and approximated normal distribution of variables for Pearson correlations. The Shapiro–Wilk test was used to test for significant deviation from normal distribution. When assumptions were not met, measures to fulfill assumptions were employed, including the exclusion of outliers and data transformation to achieve approximated normal distribution of variables.

To test the ability to discriminate dichotomized ICP, a cutoff at ICP ≥ 15 mm Hg was chosen. Median ONSD with 95% confidence intervals (CIs) were calculated for each group and distribution of values compared using a Mann–Whitney *U*-test. Receiver operating characteristics (ROC) analysis was used to calculate associated areas under the curve (AUCs) with 95% CIs.

Abovementioned analyses were performed for the whole cohort and subsequently repeated for subgroups by whether the basal cisterns had been surgically entered.

Statistical analyses were performed using Stata (StataCorp. 2023. Stata Statistical Software: Release 18. College Station, TX: StataCorp LLC.), and *p* < 0.05 was considered significant.

## Results

### Patient Characteristics

A total of 20 patients were included in the study; 11 (55%) were male and 9 (45%) were female. The median age was 61 years (interquartile range [IQR] 48.5–68 years). Admission to Oslo University Hospital was generally early after ictus (median 0 days, IQR 0–1 days, range 0–8 days). On admission to our hospital, the median Glasgow Coma Scale score was 6.5 (IQR 4–11, range 3–14), and the median World Federation of Neurosurgical Societies score was 4.5 (IQR 4–5, range 2–5).

Regarding aneurysm location, the aSAH was from an anterior circulation aneurysm (6/20 anterior communicating artery; 5/20 middle cerebral artery; 4/20 posterior communicating artery; 1/20 internal carotid artery; 1/20 anterior cerebral artery) in 17/20 (85%) of patients and from a posterior circulation aneurysm (1/20 vertebral artery; 1/20 basilar artery; 1/20 posterior inferior cerebellar artery) in 3/20 (15%) of patients.

Aneurysm repair was performed in 19/20 patients, whereas one patient was treated conservatively. Endovascular techniques were used in 9/19 (47%) patients, surgical clipping was used in 9/19 (47%) patients, and a combined sequential surgical and endovascular approach was used in one (5%) patient. Aneurysm repair was generally initiated early after admission (median 0 days, IQR 0–1 days, range 0–6 days) both for coiling (median 0 days, IQR 0–1 days, range 0–1 days) and clipping (median 0, IQR 0–1, range 0–6 days). One patient underwent delayed surgical clipping due to the presence of significant cerebral vasospasm on admission.

### Optic Nerve Sheath Examinations

A total of 26 ultrasound examinations were performed in the 20 included patients. In all but one patient, both eyes were examined, and values averaged between eyes. In the remaining single examination in the one patient, aberrant anatomy with apparent optic nerve atrophy was noted, and therefore unilateral values were recorded. It was later discovered that the patients had known severe unilateral glaucoma explaining the finding.

Serial examinations were performed in four patients, and in these patients the number of examinations ranged between two and three. Median time of ultrasound examination was 5 days (IQR 3–10 days; range 1–16 days) after ictus and 3.5 days (IQR 2–5 days; range 1–16 days) after admission to our hospital.

At the time of ultrasound examination, all patients were on a ventilator, and the median Richmond Agitation-Sedation Scale score was − 4 (IQR − 4 to − 5). All patients had an EVD in-situ. The EVD was open against external resistance in 18/26 (69%) of examinations (median resistance 2.5 cm H_2_O, IQR 0–5 cm H_2_O, range 0–10 cm H_2_O) and was closed in the remaining examinations.

At time of ultrasound examination, patients had been treated by surgical clipping in 12/26 (46%) instances, by coiling in 11/26 (42%) instances, by both coiling and clipping in 1/26 (4%), and in 2/26 (8%) instances the aneurysm had not been repaired.

In 8/26 (31%) instances, the basal cisterns had been surgically entered before ultrasound examination. These entrances to the basal cisterns were more specifically related to clip ligation of anterior communicating artery aneurysms (four patients, four examinations), obtaining proximal control for a middle cerebral artery aneurysm (one patient, three examinations) and clip ligation of a posterior communicating artery aneurysm (one patient, one examination). The median time from surgical entrance of the basal cisterns to ultrasound examination was 2.5 days (IQR 1–3.5 days, range 1–10 days).

At time of ultrasound examination, ICP was actively managed in 24/26 of instances, while in the remaining two instances (two patients), active management of the neurological condition, including ICP management, had been withdrawn.

### Invasive ICP and CPP

Invasive mean ICP values recorded at the time of examination ranged between 0 and 88 mm Hg (median 9 mm Hg; IQR 4–17 mm Hg). In all but two examinations, the ICP ranged between 0 and 22 mm Hg. The two outliers (ICP 59 mm Hg and 88 mm Hg) corresponded to examinations performed in the two patients in which active management had been withdrawn. In these patients, CPP was near zero (− 4 mm Hg and 2 mm Hg), indicating tamponade or near-tamponade. In the remaining actively managed patients, the CPP ranged between 64 and 100 mm Hg (median 75 mm Hg, IQR 71–85 mm Hg).

Regarding the distribution of ICP values, a Shapiro–Wilk test indicated that the distribution of values significantly deviated from normality (W 0.62, *p* < 0.001). Graphically, this deviation from normality appeared clearly related to the two outliers (Supplemental Fig. 1). Both exclusion of the outliers and natural log transformation of ICP (ln[ICP]) (one missing value due to ICP being 0 mm Hg) led to distributions approaching normality with Shapiro–Wilk tests indicating that distributions no longer significantly deviated from normality (W = 0.98; *p* = 0.93 and W = 0.92; *p* = 0.055 respectively). QQ-plots showing the distributions are shown in Supplemental Fig. 1. Correlation and regression results after exclusion of the two outliers are reported in the following sections while results after log transformation are reported in Supplemental Fig. 2.

**Fig. 2:**
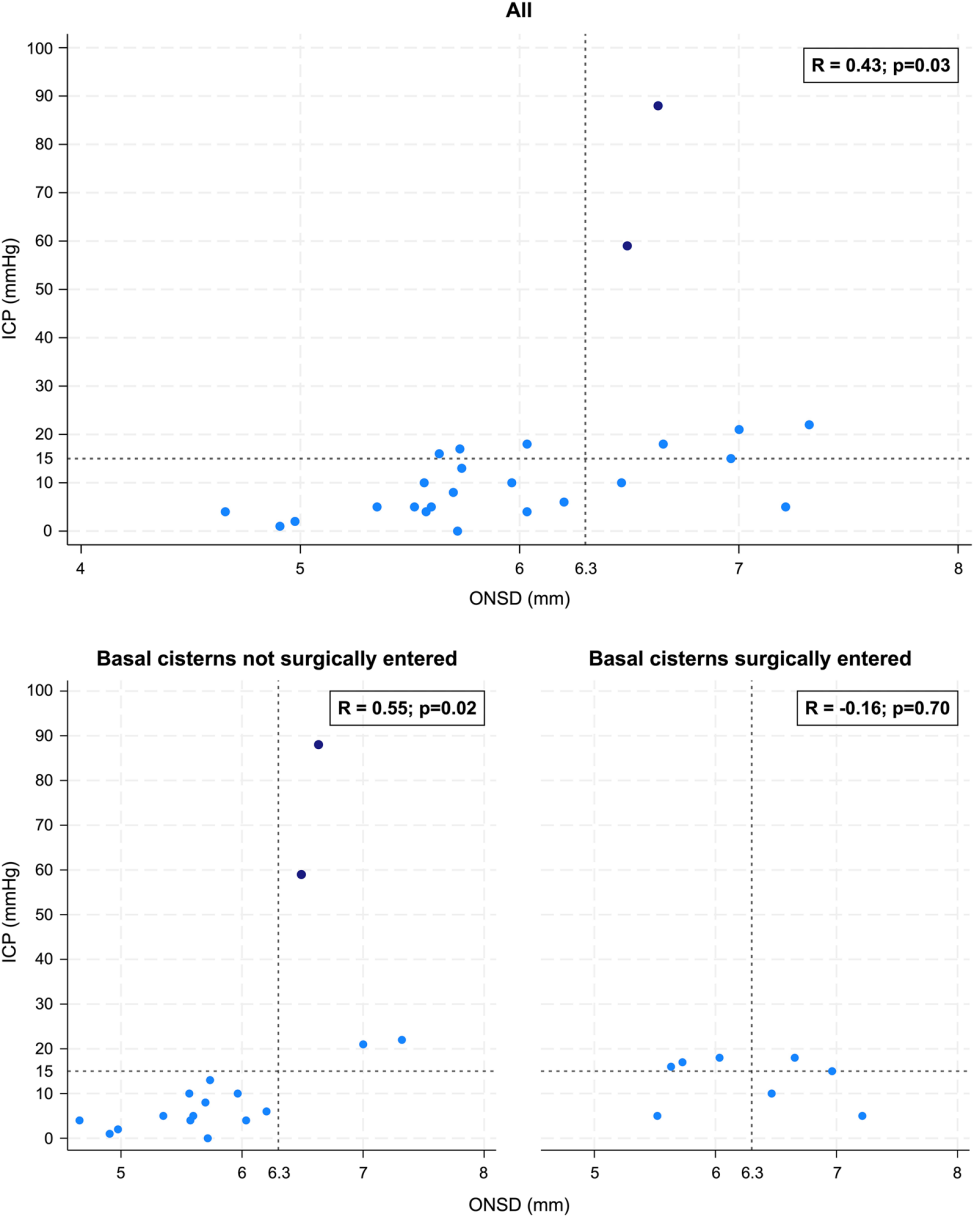
Association between ONSD and ICP in the whole cohort and in subgroups by surgical entrance to the basal cisterns. Scatterplots showing observations with correlations coefficients *R*. Top: whole cohort. Bottom left: basal cisterns not surgically entered before ultrasound examination. Bottom right: basal cisterns surgically entered before ultrasound examination. Blue dots indicate observations in patients who were under active management, whereas navy dots indicate observations in patients in whom active management, including ICP and CPP management, had been withdrawn. ICP, intracranial pressure, ONSD, optic nerve sheath diameter, CPP, cerebral perfusion pressure

### ONSD

Automatically measured ONSD ranged between 4.66 mm and 7.32 mm (median 5.82 mm, IQR 5.57–6.49 mm).

Generally, there was a very good agreement between the two eyes in those with bilateral examinations (*n* = 25), with a median difference of 0.07 mm (IQR − 0.18 to 0.32 mm; range − 1.17 to 0.87 mm).

Regarding the distribution of ONSD, values approximated normal distribution (Supplemental Fig. 1) and a Shapiro–Wilk test indicated no significant deviation from normality (W = 0.96; *p* = 0.49).

### Association, Correlation, and Linear Regression

There was an association between ONSD and ICP with a positive correlation (Pearson R 0.43; *p* = 0.03). Scatter plots of ONSD against ICP values are shown in Fig. 2.

In subgroups by whether the basal cisterns had been surgically entered before ultrasound examination, a stronger correlation (Pearson *R* = 0.55, *p* = 0.01) was seen in those in whom the basal cisterns had not been surgically entered (*n* = 18 examinations) as compared with the whole cohort. Conversely, in those in whom the basal cisterns had been surgically entered (*n* = 8), no association between ONSD and ICP was observed (Pearson *R* = − 0.16; *p* = 0.70) (Fig. 2).

**Fig. 3:**
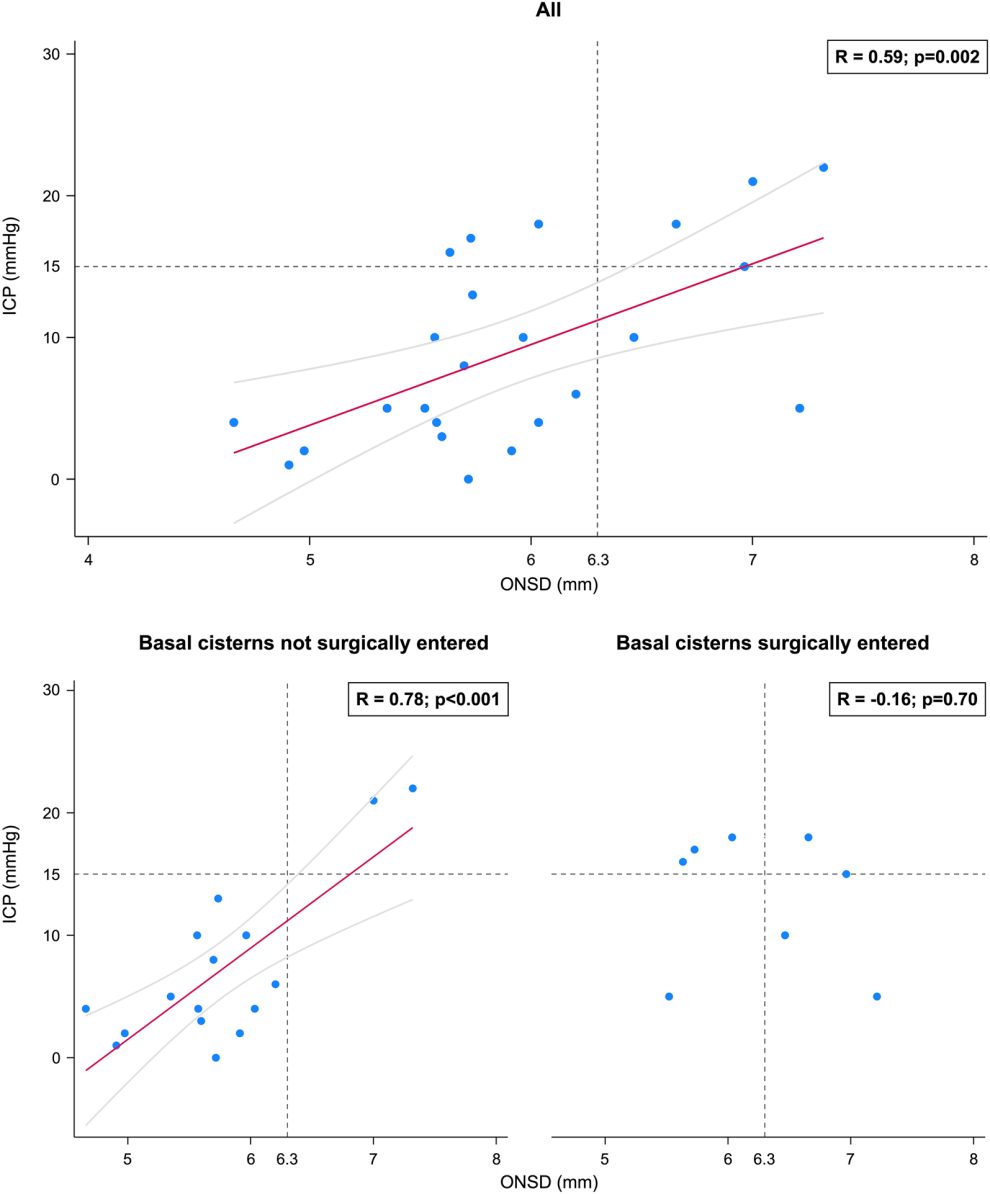
Association between ONSD and ICP in patients under active management (exclusion of ICP outliers). Scatterplots showing observations with correlation coefficients *R* and associated regression lines with 95% confidence intervals. Top: whole cohort under active management. Bottom left: basal cisterns not surgically entered before ultrasound examination. Bottom right: basal cisterns surgically entered before ultrasound examination. ICP, intracranial pressure, ONSD, optic nerve sheath diameter

When restricting observations to those in whom patients were under active management, thereby excluding the two ICP outliers, correlation increased to Pearson *R* = 0.59; *p* = 0.002 for the whole cohort (*n* = 24 examinations) and to *R* = 0.78; *p* < 0.001 for the subgroup where the basal cisterns had not been entered (*n* = 16 examinations) (Fig. 3). When fitting the same observations in linear regression models, results showed that ONSD significantly predicted ICP, both in the whole cohort (*R*^2^ = 0.35, F(1, 22) = 11.93, *p* = 0.002), with the equation ICP_Predicted_ = − 24.68 + 5.70 × ONSD, and in the subgroup where the basal cisterns had not been entered (*R*^2^ = 0.60, F(1, 14) = 21.25, *p* < 0.001), with the equation ICP_Predicted_ = − 35.76 + 7.45 × ONSD. Regression lines with 95% CIs are shown in Fig. 3.

### Ability to Discriminate Dichotomized ICP

**Fig. 4:**
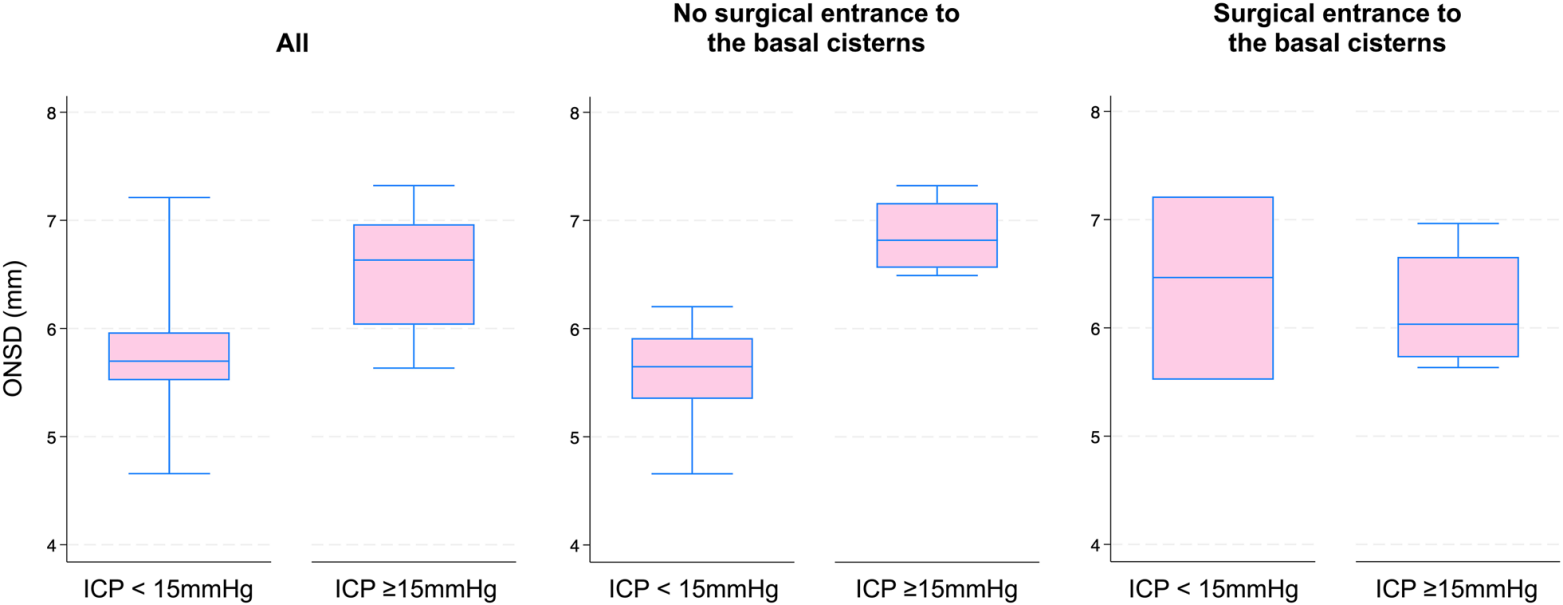
Distribution of ONSD values for ICP dichotomized at ≥ 15 mm Hg. Box plots showing median and interquartile range, and whiskers showing range. Left: whole cohort. Middle: basal cisterns not surgically entered before ultrasound examination. Right: basal cisterns surgically entered before ultrasound examination. ICP, intracranial pressure, ONSD, optic nerve sheath diameter

Analysis by dichotomization at ICP ≥ 15 mm Hg showed a significant difference in the distribution of ONSD values between the groups (high ICP *n* = 9: median ONSD 6.63 mm, 95% CI 5.75–7.00; and low ICP *n* = 17: median ONSD 5.70, 95% CI 5.52–5.96; *p* = 0.005). In subgroups by whether the basal cisterns had been surgically entered, a significant difference in ONSD was withheld in those in whom the basal cisterns had not been surgically entered (high ICP *n* = 4: median ONSD 6.82 mm,,95% CI 6.49–7.32 mm; and low ICP *n* = 14: median ONSD 5.65 mm, 95% CI 5.29–5.92 mm; *p* = 0.003), whereas in those in whom the basal cisterns had been surgically entered, no difference was seen (high ICP *n* = 5: median ONSD 6.03 mm, 95% CI 5.63–6.96 mm; and low ICP *n* = 3: median ONSD 6.46 mm, 95% CI 5.52–7.21; *p* = 0.88). Box plots graphically showing median, IQR, and ranges are displayed in Fig. 4.

**Fig. 5:**
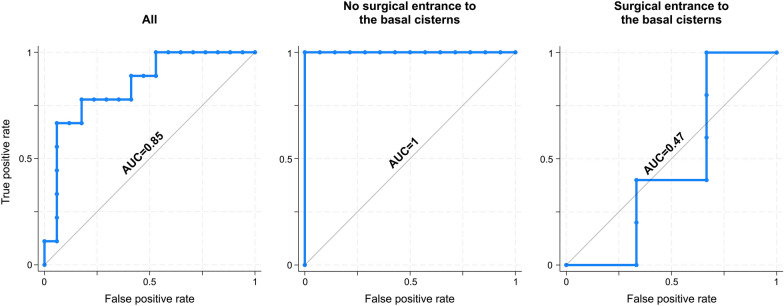
Ability to discriminate dichotomized ICP. Receiver operating characteristics curves. ICP cutoff at ≥ 15 mm Hg. Left: whole cohort. Middle: basal cisterns not surgically entered before ultrasound examination. Right: basal cisterns surgically entered before ultrasound examination. AUC, area under the curve, ICP, intracranial pressure

ROC analysis gave an AUC of 0.84 (95% CI 0.65–0.96) for the whole cohort. In subgroups by whether the basal cisterns had been surgically entered, ROC analysis revealed a perfect discriminatory ability (AUC 1, 95% CI 0.81–1) in those in whom the basal cisterns had not been surgically entered (*n* = 18 examinations) and no discriminatory ability (AUC 0.47, 95% CI 0.16–0.84) in those in whom the basal cisterns had been surgically entered. ROC curves are shown in Fig. 5.

## Discussion

In this study, we demonstrate that automatically measured ONSD correlates with ICP in patients with aSAH. Consistent with this, we further show that ONSD is able to discriminate dichotomized ICP. The results indicate that the association between ONSD and ICP is driven by a stronger association in patients in whom the basal cisterns had not been entered surgically before optic nerve sheath examination, whereas no association was observed in patients in whom basal cisterns had been entered surgically before optic nerve sheath examination. Consistent with this, ONSD showed a good overall ability to discriminate dichotomized ICP, whereas in the subgroup in whom the basal cisterns had not been entered surgically, a perfect discriminatory ability was observed in the current cohort. Again, no discriminatory ability was seen in those in whom the basal cisterns had been surgically entered before optic nerve sheath examination.

Our study has several important limitations. First, it is a single-center study and as such, findings require verification. Second, the overall number of observations is limited, which has implications for power in the analyses, especially so in the subgroup analyses. Third, the number of observations in the high ICP range is limited. This is related to the patient group studied, as a main goal of neurointensive care of patients with aSAH is to manage high ICP. Although the typical treatment threshold in aSAH is 20 mm Hg, what constitutes “normal ICP” is by most considered lower, and limits of 10 mm Hg and 15 mm Hg have been suggested [16–18]. Moreover, the threshold associated with harm on a group level is not known with certainty [19], and data have suggested that sustained ICP > 10 mm Hg [18] may be associated with worse outcomes in ICP-monitored neurocritically ill patients. To explore ONSD’s ability to discriminate ICP, we chose to dichotomize ICP at ≥ 15 mm Hg to maintain a relative group size balance. Although this dichotomization does not reflect the typical treatment threshold used, we believe that using this cutoff still provides valid information on the discriminatory ability of ONSD. Fourth, we observed that the ICP values did not distribute along approximate normality, which implies that the *p*-values of the Pearson correlations in which outliers were not excluded should be interpreted with some caution. The distribution of the observed ICP values may be specific to the current data set but may also be seen in context with the nonlinear pressure–volume curve relating to the Monro-Kellie doctrine [20]. Statistically, we explored both natural log transformation of ICP and exclusion of the two ICP outliers. Both approaches yielded approximated normal distributions of ICP, and both yielded very similar end results in terms of strength of correlation between ONSD and ICP. Because the two outliers differed qualitatively from the rest of the observations in that active management had been withdrawn and CPP was near zero, indicating tamponade, we chose exclusion of the outliers, as this approach was considered better rooted in physiological factors and not just statistical ones. However, natural log transformation of ICP did yield a more linear relationship between ONSD and ICP for all ICP values. Whether this is a trait specific to the current data set or holds true also in external data sets warrants further consideration in our opinion.

Nonetheless, in toto, our study does show a clear association between ultrasonically measured ONSD and invasively measured ICP and is, to the best of our knowledge, the first study of a cohort of pure aSAH patients to show such an association. In contrast to the current findings, several previous studies have reported no association between ultrasonically measured ONSD and invasively measured ICP in aSAH [8, 9]. This lack of association has been hypothesized to be explained by the abrupt and substantial ICP rise associated with the initial bleed leading to irreversible distention of the optic nerve sheath and thereby precluding its subsequent sensitivity to change in ICP [8–10], and alternatively by the trabeculated perioptic subarachnoid space that may be clotted by blood products, thereby losing its ability to propagate CSF pressures along the nerve [8, 11]. The findings of the current study do not support these hypotheses.

Physiologically, the data point to two interesting phenomena. First, the data clearly indicate that there is an association between ONSD and ICP, but that this association seems to be completely lost if the basal cisterns have been surgically entered before optic nerve sheath examination. The reason for this remains undetermined, but one can hypothesize that the opening of arachnoid CSF divisions in close proximity to the entrance of the perioptic cisterns may physiologically disturb the baseline premises of the correlation of ONSD to ICP by creating a CSF “highway” to the entrance of the perioptic cisterns, thereby altering the propagation of pressures into the perioptic space. Second, the relationship between ONSD and ICP seem to approximate a linear relationship except for in the two outliers with very high ICPs. At the same time, these two examinations also critically differ from the remaining in that they are from patients in whom active management had been withdrawn. CPP values were near null, hence they were in state of brain tamponade or near-tamponade. Based on this, one can hypothesize that another baseline premise of a linear relationship between ONSD and ICP is an intact CSF pulsation propagated from cerebral blood flow and that when this pulsation ceases when a state of tamponade approaches, ONSD values drop off the linear relationship. Clinically, this is of less importance, as the application of ONSD must be to detect raised ICP before a state of tamponade develops. Both of these physiological observations, albeit interesting, obviously remain hypothesis-generating only and need further examination.

This study is the first to employ real-time automated measurements of ONSD at the bedside. As such, the present results indicate that the automated measurements do indeed work when the software is coupled to an ultrasound machine in its intended point-of-care environment. Theoretically, measurement of ONSD using ultrasound can be viewed as quite a standardized technique. It has, however, been shown that considerable variation in the interpretation of the ultrasonographic representation of the sheath exists between studies [21], illustrating that the method, like most other point-of-care ultrasound, is user dependent. Automatization of measurements holds potential in alleviating some of this user dependency. If user dependency can be reduced, this may in turn increase the practical availability of ONSD. The potential uses of ONSD are multiple, and in a reality of finite health care resources, this may ultimately promise better care for neurocritically ill patients.

## Conclusions

Automatically measured ONSD correlated well with ICP and displayed a perfect discriminatory ability in patients with aSAH in whom the basal cisterns had not been entered surgically before ultrasound examination and may be a clinically valuable noninvasive marker of ICP in these patients. Caution should be exercised in using ONSD in patients in whom the basal cisterns have been entered surgically before ONSD measurements, as no association was observed in this subgroup of patients.

## Supplementary Information

Below is the link to the electronic supplementary material.

Supplemental Figure 1: Distribution of ONSD, ICP, log transformed ICP (ln(ICP)) and ICP in patients under active management (ICP_Outliers excluded_). QQ-plots showing distribution of values. Shapiro-Wilk tests indicated that the distribution of ICP deviated significantly from normality (W 0.62; p<0.001), while that of ONSD did not (W 0.96; p=0.49). After log-transformation of ICP or after exclusion of the two ICP outliers, the distributions approached normality and repeated Shapiro-Wilk tests no longer indicated significant deviation from normality (W 0.98; p=0.93 and W 0.92; p=0.055 respectively) (JPG 3172 KB)

Supplemental Figure 2: Association between ONSD and ln (ICP). Scatterplots showing observations with correlation coefficients R, regression lines with 95% CIs and results from linear regressions model fitment. Top: whole cohort. Bottom left: basal cisterns not surgically entered prior to ultrasound examination. Bottom right: basal cisterns surgically entered prior to ultrasound examination. Blue dots indicate observations in patients who were under active management, while navy dots indicate observations in patients in whom active management, including ICP and CPP management, had been withdrawn (JPG 3084 KB)
